# Latent profiles and transitions of acute psychological stress in patients with acute myocardial infarction after percutaneous coronary intervention and their association with cardiac rehabilitation participation

**DOI:** 10.3389/fpubh.2026.1939161

**Published:** 2026-09-15

**Authors:** Fei Shen, Jingzhi Luo, Jun Wu, Lin Xing

**Affiliations:** 1 Department of Cardiology, Wuxi People's Hospital Affiliated to Nanjing Medical University, Wuxi, China; 2 Department of Cardiology, No. 904 Hospital of the Joint Logistics Support Force, PLA, Wuxi, China; 3 Department of Cardiology, The First Affiliated Hospital of Soochow University, Suzhou, China

**Keywords:** acute myocardial infarction, acute psychological stress, cardiac rehabilitation participation, latent profile analysis, latent transition analysis, nursing, three-stage dynamic evolution

## Abstract

**Aim:**

To explore the dynamic transitions of latent profiles of acute psychological stress in patients with acute myocardial infarction across three time points and their association with cardiac rehabilitation participation, and to provide new insights for improving cardiac rehabilitation participation in patients with acute myocardial infarction.

**Design:**

This study was a prospective longitudinal cohort study.

**Methods:**

The convenience sampling method was used to select 262 patients with acute myocardial infarction as research subjects. Measurements were conducted three times using the Stanford Acute Stress Reaction Questionnaire and the cardiac rehabilitation scale: at T1 (after PCI when the patient’s condition was stabilized), at T2 (1 month after PCI), and at T3 (3 months after PCI). Latent profile analysis (LPA) was used to identify heterogeneous subgroups of acute psychological stress at each time point. Latent Transition Analysis (LTA) was used to characterize the dynamic transition patterns over time. Furthermore, a multivariate Logistic regression model was applied to examine the association between different stress evolution trajectories and low cardiac rehabilitation participation.

**Results:**

A total of 248 valid questionnaires were collected. The prevalence of severe acute stress symptoms at T1, T2 and T3 was 58.87, 29.84 and 18.15%, respectively. At the three time points of acute psychological stress measurement, three latent profiles were identified: low psychological stress group (C1), moderate psychological stress group (C2) and high psychological stress group (C3). Latent transition analysis identified six main evolution types: 28.23% for C1 → C1 → C1, 18.55% for C2 → C2 → C2, 22.18% for C3 → C3 → C3, 14.52% for the remitting type (C3 → C2 → C1), 8.06% for the fluctuating type (C2 → C3 → C2) and 8.47% for the worsening type (C1 → C2 → C3). Compared with the persistently low-stress type, AMI patients with the persistently moderate-stress type (OR = 2.845, 95% CI: 1.542–5.248), persistently high-stress type (OR = 10.237, 95% CI: 5.128–20.415), remitting type (OR = 2.156, 95% CI: 1.298–3.582), fluctuating type (OR = 4.521, 95% CI: 2.567–7.965), and worsening type (OR = 6.834, 95% CI: 3.245–14.385) had significantly higher odds of low cardiac rehabilitation participation (all *p* < 0.05).

**Conclusion:**

Acute psychological stress evolution trajectories are significantly associated with cardiac rehabilitation participation among patients with acute myocardial infarction. There are complex two-way transitions between stress latent profiles, and early clinical identification and continuous psychological intervention are associated with improved patients’ cardiac rehabilitation participation levels.

## Introduction

Acute myocardial infarction (AMI) is a major global public health problem characterized by high disability and mortality rates, and its disease burden continues to increase (1, 2). Global data indicate that its 30-day mortality rate ranges from 5 to 15% (3). According to the Report on Cardiovascular Health and Diseases in China, the morbidity and mortality of AMI in China continue to rise, with the incidence reaching 82.76 per 100,000 population. It is estimated that an additional 21 million acute coronary events will occur over the next 20 years, making the overall situation very serious (4, 5). In particular, China lags behind developed countries in both the proportion and timeliness of reperfusion therapy for AMI. Against this background, it is extremely important to explore interventions that can improve the prognosis of AMI patients (6).

Severe acute stress symptoms is a psychological stress that occurs within the first month after experiencing or witnessing a traumatic event (such as a life-threatening illness), primarily characterized by dissociative symptoms, avoidance of trauma-related stimuli, re-experiencing, anxiety, or hyperarousal (7, 8). Studies indicate that over 50% of AMI inpatients experience anxiety or acute stress symptoms, and 20–30% remain affected months after discharge (9), which is significantly higher than in the general population. Acute psychological stress significantly increases the stress of major adverse cardiovascular events (MACE): it raises the risk of death by 46% and the risk of recurrent myocardial infarction by 28–60%. In addition, depressed patients under stress often develop avoidance behaviors such as loss of interest, which reduces their adherence to rehabilitation programs and leads to low participation in recovery, seriously affecting patients’ long-term prognosis (10, 11).

Previous studies have mostly adopted a cross-sectional design to explore the association between psychological stress and rehabilitation participation; however, such studies have notable limitations, failing to capture the dynamic evolution trajectories of acute psychological stress after PCI, making it difficult to identify the characteristics of different stress subgroups, and further unable to reveal the association between stress trajectories and cardiac rehabilitation participation, thus offering limited clinical guidance value.

Building on this, the present study aimed to employ a prospective longitudinal cohort design to conduct repeated assessments at the acute phase after PCI, 1 month post-PCI, and 3 months post-PCI in patients with AMI. It seeks to explore the heterogeneous latent profiles of acute psychological stress and their dynamic transition trajectories over time, and to examine their association with cardiac rehabilitation participation, thereby providing a basis for implementing whole-process and comprehensive clinical management.

## Subjects and methods

### Subjects

Using convenience sampling, from May to September 2025, trained researchers at the cardiology wards of three tertiary A hospitals in Jiangsu Province reviewed the daily admission lists to screen for eligible patients with acute myocardial infarction (AMI) who had undergone percutaneous coronary intervention (PCI) based on the inclusion and exclusion criteria. Eligible patients were invited to participate in the study after their condition stabilized. A total of 262 patients were enrolled and completed follow-up assessments at three time points: baseline (T1), 1 month post-PCI (T2), and 3 months post-PCI (T3) (Figures 1–4).

**Figure 1:**
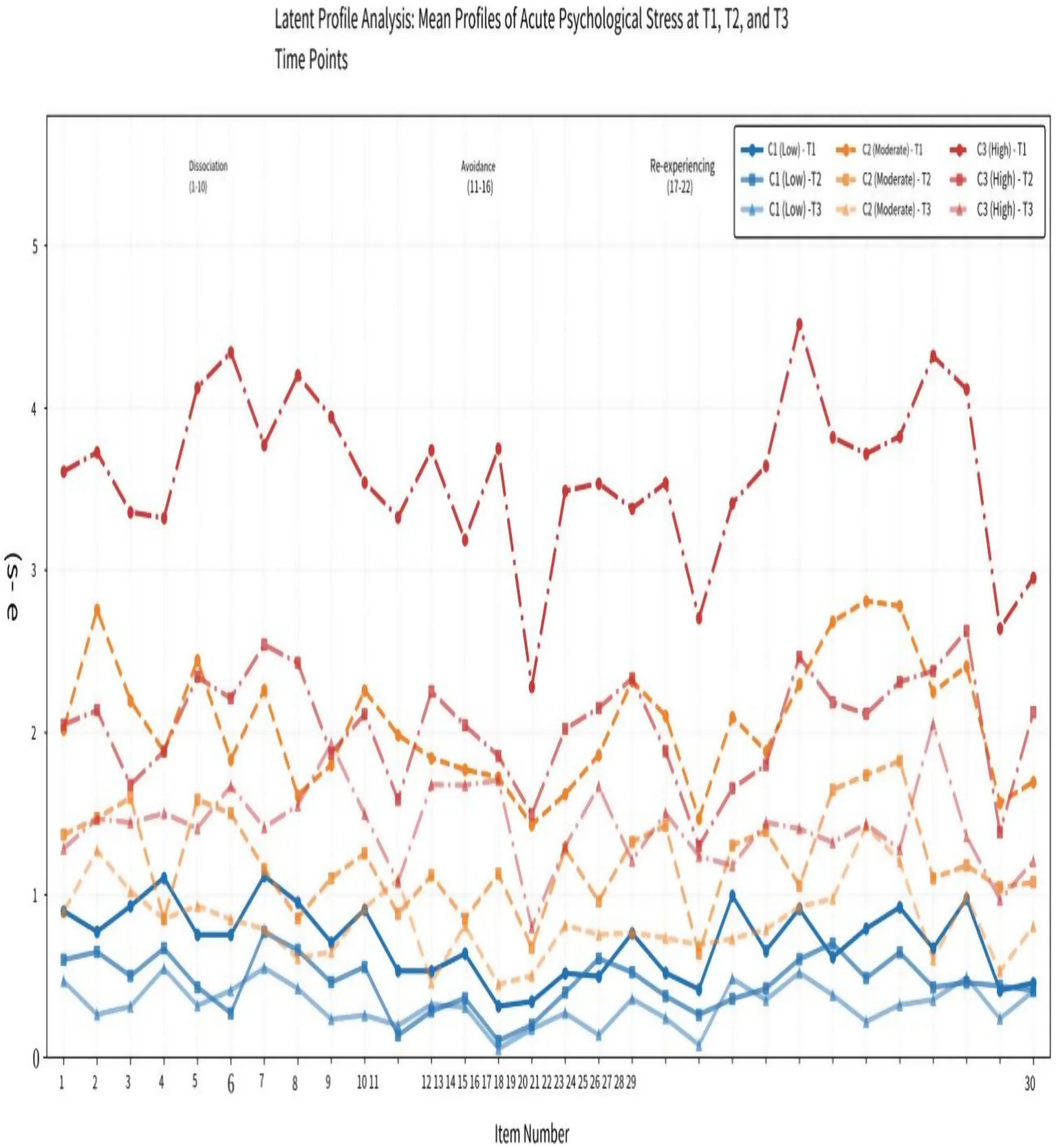
In patients with acute myocardial infarction (mi) of PCI postoperative acute psychological stress latent profile analysis, three time points section average figure.

**Figure 2:**
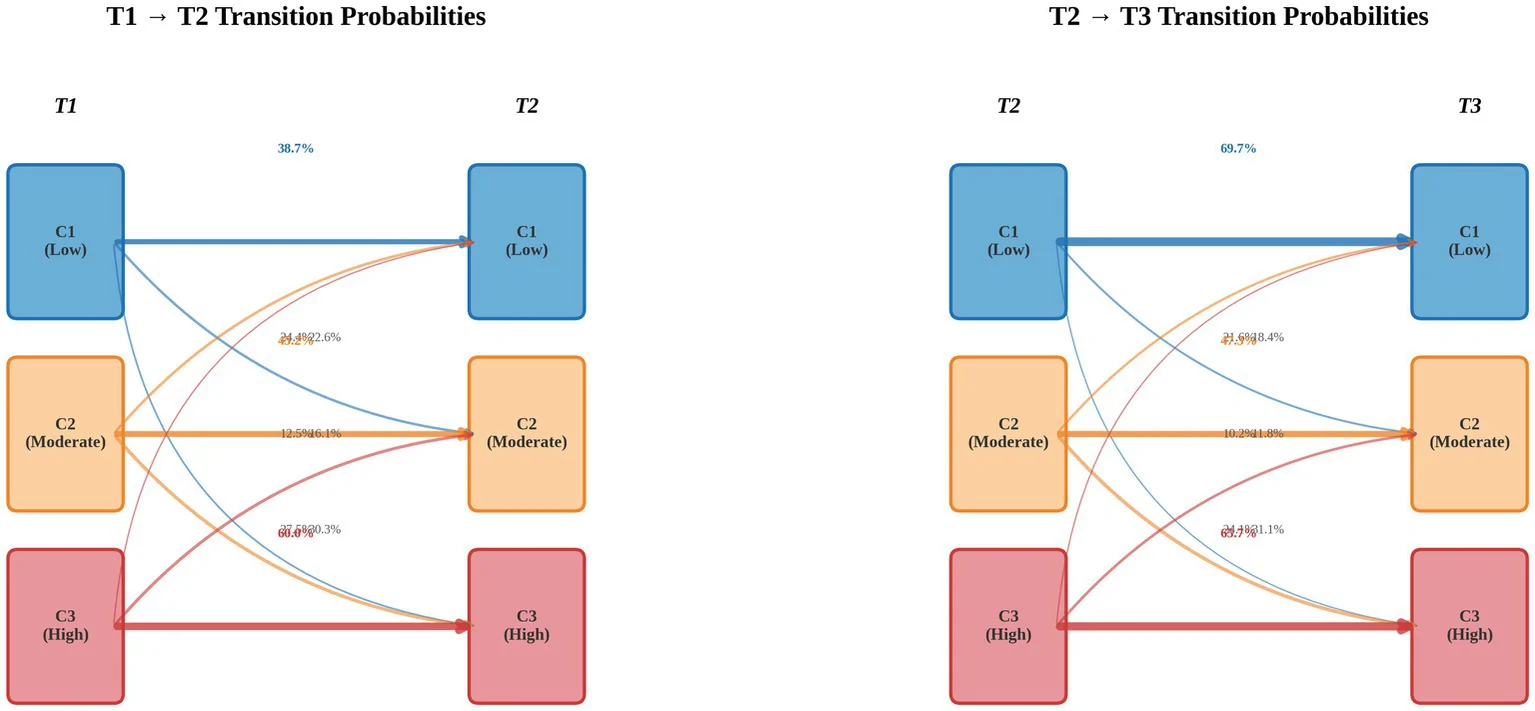
Matrix of transition probabilities across time for latent profiles of acute psychological stress in AMI patients.

**Figure 3:**
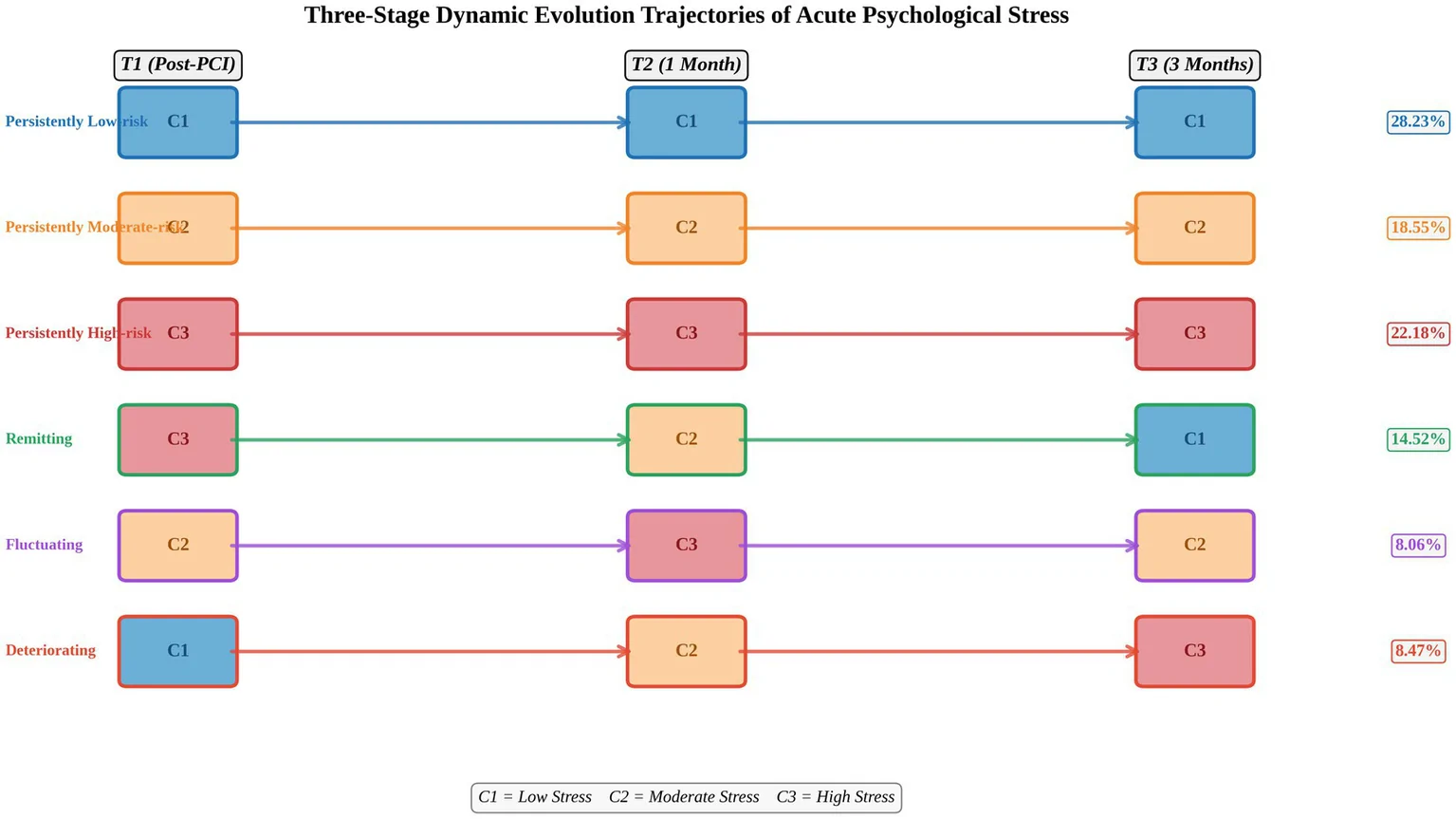
The three-stage dynamic evolution trajectory of acute psychological stress in patients with acute myocardial infarction.

**Figure 4:**
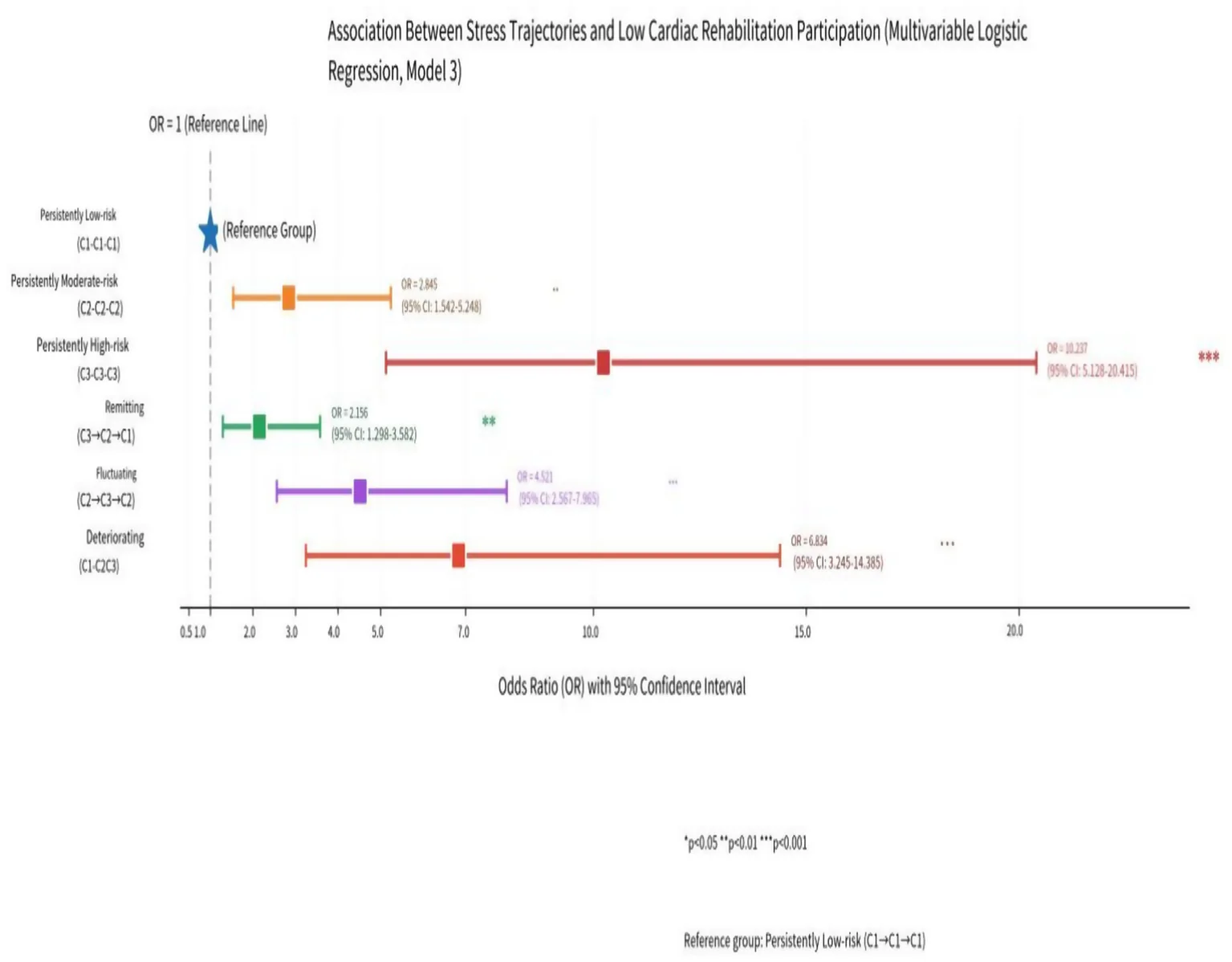
Association between different trajectories of psychological stress evolution and the stress of low participation in cardiac rehabilitation (forest plot).

Inclusion criteria: patients with confirmed acute myocardial infarction, aged 18 years and older; first-time myocardial infarction and treated with PCI; all patients had basic communication skills and signed informed consent.

Exclusion criteria: pre-existing movement disorders, psychological disorders or mental illness; death or failure to complete 3 longitudinal measurements for other reasons during follow-up.

Based on the repeated-measures sample size formula (12) with *α* = 0.05 and 1 − *β* = 0.9, this study conducted a pilot study prior to the formal investigation. Using convenience sampling, 30 AMI patients who underwent PCI at a tertiary A hospital in Jiangsu Province in April 2025 were selected and assessed three times using the same instruments at the same three time points to obtain the parameters required for sample size calculation. Data from these 30 patients were used to estimate the following parameters: *K* = 3 (three repeated measurements), measurement error variance *σ*^2^ = 124.667, conditional correlation coefficient *ρc* = 0.724, and between-individual variance *σ*^2^*μ* = 360.533. Based on these estimates, a minimum of 179 participants was required. Accounting for a 10% attrition rate in this longitudinal survey, the minimum sample size was set at *n* = 179 ÷ (1–10%) = 199. A total of 262 patients were ultimately enrolled, primarily considering the feasibility of multicenter convenience sampling and a potentially higher-than-expected attrition rate, to ensure that the final effective sample size would meet the statistical requirements. The three repeated measurements from the 30 pilot study patients were used to estimate the aforementioned variances and correlation coefficients, and this sample size was sufficient for parameter estimation in the pilot phase. This clinical investigation was conducted after obtaining approval from the ethics committees of the three participating hospitals (approval numbers: 2024-060052, HDPH2024060031, sudafuyi zi-2024060135). All subjects volunteered to participate in this study and signed informed consent.

### Survey instrument

#### General information questionnaire

The general information questionnaire was designed by the researchers, collecting information including age, gender, marital status, education level, occupation, family income, number of comorbidities, number of stents, left ventricular ejection fraction, and cardiac function classification, among other variables.

#### Stanford acute stress reaction questionnaire (SASRQ)

The SASRQ was translated and validated in Chinese by scholar Jia Fujun et al. (13). The Chinese version of the SASRQ consists of 5 dimensions: dissociative symptoms (10 items), avoidance symptoms of traumatic events (6 items), repeated re-experiencing of traumatic events (6 items), anxiety or increased arousal symptoms (6 items), and social function impairment (2 items), totaling 30 items. Each item is scored from 0 to 5 points, with an overall total score ranging from 0 to 150 points. According to the original scale developers, a total score ≥ 57 indicates severe acute stress symptoms (13). When completing the questionnaire, patients were instructed to regard their “acute myocardial infarction event and PCI procedure” as the traumatic event. The Cronbach’s *α* coefficients of the scale at T1, T2, and T3 in this study were 0.842, 0.833, and 0.821 (Cronbach’s *α* coefficient, respectively).

#### Cardiac rehabilitation scale

This scale was developed by Wang Junhong et al. (14), and includes 3 dimensions: process anxiety (7 items), outcome anxiety (5 items), and autonomy (6 items), totaling 18 items. Each item is scored from 0 to 4 points, ranging from “strongly disagree” to “strongly agree,” items in the process anxiety and result anxiety dimensions are reverse-scored, while items in the autonomy dimension are forward-scored. The total score ranges from 0 to 72, with higher scores indicating a greater level of patient participation in cardiac rehabilitation. A score of 0–36 indicates a relatively low level of rehabilitation participation. The Cronbach’s *α* coefficients of the scale at the three measurement time points in this study were 0.810, 0.853, and 0.828.

### Data collection methods

Before the survey, all investigators involved in the data collection were trained. The questionnaire survey was conducted after obtaining consent from both the hospitals and the patients. The general information questionnaire was extracted from the patients’ medical records. Data from the Acute Psychological Stress Scale (SASRQ) and the Cardiac Rehabilitation Participation Scale (CRS) were collected at three time points: after PCI when the patient’s condition was stabilized (specifically defined as stable vital signs for at least 24 h post-operation, no recurrent chest pain or severe complications, and confirmation of clinical stability by the attending physician, usually within 3–7 days after surgery) (T1), 1 month after PCI (T2), and 3 months after PCI (T3). Data at T1 were collected via face-to-face interviews in the cardiology ward, while data at T2 and T3 were collected through outpatient follow-up visits or telephone follow-ups combined with online questionnaires. To minimize potential bias arising from different data collection modes, the online questionnaires used the same instructions and item wording as the paper versions, and trained researchers provided unified explanations for any queries during the telephone follow-ups to ensure consistent comprehension of the items among patients.

In addition, during the study observation period (T1–T3), all three hospitals strictly followed the Chinese Guidelines for the Diagnosis and Treatment of Acute Myocardial Infarction and hospital nursing routines to provide standardized discharge guidance and cardiac rehabilitation health education for admitted AMI patients. The specific content included: (1) Medication guidance: emphasizing the standardized use of dual antiplatelet agents, statins, etc., and monitoring for adverse reactions; (2) Exercise rehabilitation guidance: based on the patient’s cardiac function classification, rehabilitation nurses demonstrated and guided home-based exercise programs (e.g., moderate-intensity aerobic exercise 3–5 times a week, 30 min each session); (3) Lifestyle interventions: smoking cessation, alcohol restriction, low-salt and low-fat diet, weight control, etc.; (4) Psychological support: before discharge and during follow-up outpatient visits, cardiology healthcare providers conducted brief psychological assessments, informed patients about the commonality of postoperative emotional reactions such as anxiety and depression, and provided relaxation training advice and guidance on family support. None of the three centers provided additional, systematic cognitive behavioral therapy or mandatory cardiac rehabilitation programs specifically for the patients in this study during the observation period. All psychosocial support and rehabilitation information received by the patients were entirely derived from the aforementioned routine clinical care, and the core components of the routine care protocols across the three hospitals were confirmed to be homogeneous through preliminary investigation.

A total of 262 questionnaires were distributed in this study, and 248 valid questionnaires were ultimately recovered. All 248 valid cases completed all three assessments. Missing data were primarily supplemented via telephone follow-ups. Questionnaires with a missing data rate >10% were excluded (a total of 14 were excluded: 8 due to loss to follow-up and 6 due to a missing data rate >10%). To ensure patient privacy, the survey was conducted in a confidential environment, and the questionnaires were completed anonymously.

### Statistical methods

After double data entry and verification by two researchers, the data were analyzed using SPSS 26.0 software for descriptive statistics and correlation analyses. Categorical variables were expressed as frequencies and percentages (*n*, %). Continuous variables that followed a normal distribution were expressed as mean ± standard deviation (mean ± SD). The chi-square (*χ*^2^) test was used to compare different general demographic characteristics, and Harman’s single-factor test was employed to check for common method bias.

Measurement invariance across the three time points was tested by constraining the item response probabilities of the 30 SASRQ items to be equal. The constrained model was compared to the unconstrained model using a likelihood ratio test, which was non-significant (*χ*^2^ = 45.32, df = 58, *p* = 0.862), indicating that the latent profiles are comparable across T1, T2, and T3. Therefore, the LTA was conducted under the assumption of longitudinal measurement invariance. Mplus 8.3 software was used to perform Latent Profile Analysis (LPA) and Latent Transition Analysis (LTA). LPA model fitting for 1–4 classes was conducted separately based on the scores of the 30 SASRQ items at T1, T2, and T3. The optimal number of classes was comprehensively determined based on the Akaike Information Criterion (AIC), Bayesian Information Criterion (BIC), sample-size adjusted BIC (aBIC), Entropy, Lo–Mendell–Rubin likelihood ratio test (LMR), and Bootstrap Likelihood Ratio Test (BLRT). On this basis, a three-stage LTA model was constructed to analyze the cross-time class transition patterns from T1 → T2 → T3. Theoretically, three classes at three time points could form 27 possible trajectory combinations. Based on the individual class membership probabilities estimated by the LTA model, trajectories accounting for >5% of the sample were extracted as the main trajectories, resulting in a total of six main trajectories.

Univariate and multivariate. Logistic regression analyses were used to examine the association between acute psychological stress transition patterns and cardiac rehabilitation participation. The six main stress evolution trajectories served as the independent variables, and cardiac rehabilitation participation (dichotomized using a total score of 36 as the cut-off, where 0–36 = 1 indicating low participation and 37–72 = 0 indicating high participation) served as the dependent variable, with the persistently low-stress type as the reference group. In the multivariate Logistic regression models, Model 2 was adjusted for age, sex, marital status, education level, occupation, and income; Model 3 was further adjusted for number of comorbidities, number of stents, left ventricular ejection fraction (LVEF), cardiac function classification, and T1 cardiac rehabilitation participation, based on clinical importance and variables with *p* < 0.1 in the univariate analysis. A two-tailed *p*-value < 0.05 was considered statistically significant.

## Results

### General information of the respondents

In this study, a total of 262 questionnaires were distributed, and 14 invalid questionnaires with lost follow-up or missing data rate >10% were excluded. Finally, 248 valid questionnaires were collected, with an effective response rate of 94.66%. The general characteristics of the respondents at time point T1 are shown in Table 1. Among all respondents, the proportion of older patients aged ≥60 years was the highest (51.61%); 53.23% were male, 93.55% were married, 53.23% were patients with cardiac function class I, and 47.17% were patients with LVEF>50%.

**Table 1:** General information of the subjects (*n* = 248).

Items	Categories	*n*	Percentage (%)
Age (years)	<45	39	15.73
	45–59	81	32.66
	≥60	128	51.61
Gender	Male	132	53.23
	Female	116	46.77
Education level	Junior high school or below	140	56.45
	High school/secondary school	68	27.42
	Junior college and above	40	16.13
Monthly household income (yuan)	<4,000	47	18.95
	4,000–	87	35.08
	7,000–	85	34.27
	>10,000	29	11.70
Marital status	Unmarried/divorced/widowed	16	6.45
	Married	232	93.55
Occupation	Enterprises and institutions	52	20.97
	Individual	44	17.74
	Clerk/staff	91	36.69
	Unemployed/retired	61	24.60
Number of comorbidities	<5 kinds	176	70.97
	≥5	72	29.03
Number of stents	<2	175	70.56
	≥2 pieces	73	29.44
Left ventricular ejection fraction	<40%	45	18.15
	40%–49%	86	34.68
	>50%	117	47.17
Cardiac function classification	Grade I	132	53.23
	Level II	70	28.23
	Grade III-IV	46	18.54

### Common method Bias test

In this longitudinal study, the data collected at T1, T2, and T3 time points were tested for common method bias using the Harman single factor test. The test results showed that the number of factors with eigenvalues greater than 1 at T1, T2 and T3 were 11, 10 and 9, respectively, and the proportion of variance that could be explained by the first common factor was 19.64, 25.53 and 21.37%, respectively. All these values were lower than the critical standard of 40% (15), indicating that the data obtained from the three measurements were not affected by serious common method bias (Table 2).

**Table 2:** Comparison of the prevalence of severe acute stress symptoms at T1, T2 and T3 in patients with acute myocardial infarction.

Items	Categories	*n*	T1 Severe stress [*n* (%)]	T1 *χ*^2^ (*p*)	T2 severe stress [*n* (%)]	T2 *χ*^2^ (*p*)	T3 severe stress [*n* (%)]	T3 *χ*^2^ (*p*)
Age (years)	<45	39	14 (35.90)	10.822^**^ (0.004)	7 (17.95)	4.217 (0.122)	4 (10.26)	3.854 (0.146)
	45–59	81	41 (50.62)		22 (27.16)		13 (16.05)	
	≥60	128	91 (71.09)		45 (35.16)		28 (21.88)	
Gender	Male	132	75 (56.82)	0.572 (0.450)	37 (28.03)	0.614 (0.433)	22 (16.67)	0.528 (0.468)
	Female	116	71 (61.21)		37 (31.90)		23 (19.83)	
Marital status	Unmarried/divorced/widowed	16	9 (56.25)	0.038 (0.845)	5 (31.25)	0.026 (0.872)	3 (18.75)	0.012 (0.913)
	Married	232	137 (59.05)		69 (29.74)		42 (18.10)	
Education level	Junior high school or below	140	88 (62.86)	1.942 (0.379)	46 (32.86)	2.147 (0.342)	29 (20.71)	1.876 (0.392)
	High school/secondary school	68	37 (54.41)		18 (26.47)		11 (16.18)	
	Junior college and above	40	21 (52.50)		10 (25.00)		5 (12.50)	
Monthly household income (yuan)	<4,000	47	29 (61.70)	0.896 (0.826)	20 (42.55)	9.522^*^ (0.023)	13 (27.66)	7.853^*^ (0.049)
	4,000–	87	52 (59.77)		30 (34.48)		18 (20.69)	
	7,000–	85	48 (56.47)		20 (23.53)		12 (14.12)	
	>10,000	29	17 (58.62)		4 (13.79)		2 (6.90)	
Career	Enterprises and institutions	52	28 (53.85)	2.147 (0.542)	13 (25.00)	3.214 (0.359)	8 (15.38)	2.789 (0.425)
	Individual	44	26 (59.09)		15 (34.09)		9 (20.45)	
	Clerk/staff	91	56 (61.54)		28 (30.77)		17 (18.68)	
	Unemployed/retired	61	36 (59.02)		18 (29.51)		11 (18.03)	
Number of comorbidities	<5 comorbidities	176	95 (53.98)	4.684^*^ (0.030)	46 (26.14)	3.969^*^ (0.046)	26 (14.77)	5.124^*^ (0.024)
	≥5 species	72	51 (70.83)		28 (38.89)		19 (26.39)	
Number of brackets	<2	175	96 (54.86)	3.956 ^*^ (0.047)	48 (27.43)	1.872 (0.171)	28 (16.00)	2.014 (0.156)
	≥2 pieces	73	50 (68.49)		26 (35.62)		17 (23.29)	
Left ventricular ejection fraction	<40%	45	32 (71.11)	5.217 (0.074)	22 (48.89)	15.487^**^ (<0.001)	14 (31.11)	12.356^**^ (<0.001)
	40%–49%	86	53 (61.63)		30 (34.88)		19 (22.09)	
	>50%	117	61 (52.14)		22 (18.80)		12 (10.26)	
Cardiac function classification	Grade I	132	62 (46.97)	15.121^**^ (0.001)	28 (21.21)	16.437^**^ (<0.001)	17 (12.88)	13.287^**^ (<0.001)
	Grade II	70	48 (68.57)		24 (34.29)		15 (21.43)	
	Grade III-IV	46	36 (78.26)		22 (47.83)		13 (28.26)	

^*^p < 0.05, ^**^p < 0.001; Severe acute stress symptoms was defined as SASRQ total score ≥57. T1 was after PCI/after stable condition, T2 was 1 month after PCI, and T3 was 3 months after PCI.

### Comparison of the prevalence of severe acute stress symptoms at T1, T2 and T3 in patients with acute myocardial infarction

Univariate analysis showed that age, number of comorbidities, number of stents and cardiac function classification were the factors associated with severe acute stress symptoms at T1. Among them, the incidence was 71.09% for patients aged ≥60 years and 78.26% for patients with cardiac function grade III–IV, both significantly higher than that in other subgroups (all *p* < 0.05). At T2, family monthly income, number of comorbidities, left ventricular ejection fraction, and cardiac function classification showed prominent impacts. The incidence was 42.55% for patients with family monthly income <4,000 yuan, 48.89% for those with LVEF<40, and 47.83% for those with cardiac function grade III–IV, respectively (all *p* < 0.05). At T3, the above four factors still had independent predictive effects, and the incidence was 27.66% for patients with family monthly income <4,000 yuan, 26.39% for those with comorbidities ≥5, 31.11% for those with LVEF<40 and 28.26% for those with NYHA III–IV, respectively (all *p* < 0.05). This suggests that factors associated with psychological stress have dynamic transformation characteristics during the rehabilitation process.

### Latent profile analysis of acute psychological stress in patients with acute myocardial infarction

Based on the 30-item acute psychological stress scale scores, we conducted latent profile analysis at T1, T2, and T3 to identify distinct stress response patterns. The analysis began with a 1-class model and incrementally increased the number of classes to 4, and the corresponding model fit indices are presented in Table 3. In addition to AIC, BIC, aBIC, Entropy, and LMR, we also performed the Bootstrap Likelihood Ratio Test (BLRT).

**Table 3:** Comparison of fitting parameter indexes of different latent profile models.

time	Model	AIC	BIC	aBIC	Entropy	LMR (*p*)	BLMR (*p*)	Class probability
T1	1	23923.804	23945.796	23922.064	–	–	–	–
	2	23274.773	23277.582	23277.342	0.852	0.004	<0.001	0.36/0.64
	3	**22811.262**	**22814.316**	**22806.203**	**0.891**	**0.048**	**<0.001**	**0.21/0.39/0.40**
	4	22132.177	22149.365	22148.738	0.912	0.121	0.079	0.24/0.11/0.22/0.43
T2	1	23835.341	23889.346	23827.650	–	–	–	–
	2	23108.675	23108.838	23113.712	0.875	0.000	<0.001	0.62/0.38
	3	**22607.296**	**22648.542**	**22618.638**	**0.903**	**0.039**	**<0.001**	**0.38/0.35/0.27**
	4	22004.417	22071.253	22014.156	0.922	0.135	0.125	0.27/0.17/0.22/0.34
T3	1	23742.156	23798.523	23735.891	–	–	–	–
	2	23056.234	23058.912	23061.445	0.886	0.000	<0.001	0.71/0.29
	3	**22589.123**	**22632.876**	**22595.234**	**0.912**	**0.027**	**<0.001**	**0.52/0.28/0.20**
	4	21976.543	22045.891	21988.654	0.928	0.142	<0.133	0.25/0.18/0.20/0.37

Bold lines indicate optimal model choices.

For the 4-class solution, both the LMR test (T1: *p* = 0.121; T2: *p* = 0.135; T3: *p* = 0.142) and the BLRT yielded non-significant results at all three time points (T1: LMR *p* = 0.121, BLRT *p* = 0.079; T2: LMR *p* = 0.135, BLRT *p* = 0.125; T3: LMR *p* = 0.142, BLRT *p* = 0.133), indicating that it did not significantly improve model fit compared with the 3-class model. Although the 4-class solution showed slightly higher Entropy values (T1: 0.912; T2: 0.922; T3: 0.928) and lower information criteria, it produced a very small, clinically ambiguous subclass (e.g., only 11% at T1, labeled as the fourth class), which lacked substantive interpretability. After comprehensive evaluation of multiple indicators, the 3-class model was identified as the optimal solution at all three time points: for T1’s 3-class model, Entropy = 0.891, LMR test *p* = 0.048, BLRT *p* = 0.045; for T2, Entropy = 0.903, LMR test *p* = 0.039, BLRT *p* = 0.032; for T3, Entropy = 0.912, LMR test *p* = 0.027, BLRT *p* = 0.021. All results met the requirements for classification accuracy. Based on the score characteristics of each class, Class 1 was labeled the “low psychological stress group (C1)”, Class 2 was labeled the “moderate psychological stress group (C2)”, and Class 3 was labeled the “high psychological stress group (C3)”.

### Three-phase latent transition analysis of acute psychological stress in patients with acute myocardial infarction

Before conducting the LTA, we established the comparability of the latent profiles across time by testing longitudinal measurement invariance (see Statistical Methods). The results supported that the three-class solution represented the same underlying construct at T1, T2, and T3, justifying the subsequent transition analysis.

This study used three-stage latent transition analysis to investigate the dynamic changes of acute psychological stress profiles in patients with acute myocardial infarction at the T1, T2, and T3 time points.

The T1 → T2 transition matrix showed that 38.7% of C1 patients remained in the original category, 45.2% transitioned from C1 to C2, and 16.1% transitioned from C1 to C3; 45.2% of C2 patients transitioned to C1, 24.4% remained in C2, and 30.3% transitioned to C3; 12.5% of C3 patients transitioned to C1, 27.5% transitioned to C2, and 60.0% remained in C3.

The T2 → T3 transition matrix showed that 69.7% of C1 patients remained in the original category, 18.4% transitioned to C2, and 11.8% transitioned to C3; 21.6% of C2 patients transitioned to C1, 47.3% remained in C2, and 31.1% transitioned to C3; 10.2% of C3 patients transitioned to C1, 24.1% transitioned to C2, and 65.7% remained in C3.

Based on the three-stage transition types, theoretically, the 3 classes across the 3 time points could form 27 possible trajectory combinations. Based on the individual class membership probabilities estimated by the LTA model, trajectories accounting for >5% of the sample were extracted as the main trajectories, yielding 6 main trajectories in total: 28.23% persistently low-stress type (C1 → C1 → C1), 18.55% persistently moderate-stress type (C2 → C2 → C2), 22.18% persistently high-stress type (C3 → C3 → C3), 14.52% remitting type (C3 → C2 → C1), 8.06% fluctuating type (C2 → C3 → C2), and 8.47% worsening type (C1 → C2 → C3). See Table 4 for details.

**Table 4:** Transition probabilities of acute psychological stress in patients with acute myocardial infarction across T1-T3 time points (*n* = 248).

Trajectory	T1 category	T2 category	T3 category	Trajectory type	Proportion (%)
Trajectory 1	C1	C1	C1	Persistently Low-stress	28.23
Trajectory 2	C2	C2	C2	Persistently Moderate-stress	18.55
Trajectory 3	C3	C3	C3	Persistently High-stress	22.18
Trajectory 4	C3	C2	C1	Remitting	14.52
Trajectory 5	C2	C3	C2	Fluctuating	8.06
Trajectory 6	C1	C2	C3	Worsening	8.47

### Association between psychological stress trajectory patterns and cardiac rehabilitation participation among patients with acute myocardial infarction

Six main stress trajectories were included as independent variables, and low cardiac rehabilitation participation (scored 0–36 points = 1, scored 37–72 points = 0) was used as the dependent variable. Multivariable Logistic regression analysis was performed with the persistently low-stress type as the control group.

In the unadjusted model (Model 1), compared with the persistently low-stress type: persistently moderate-stress type had an (OR = 4.124, 95% CI: 2.156–7.892); persistently high-stress type had an (OR = 16.237, 95% CI: 8.245–31.987); remitting type had an (OR = 3.156, 95% CI: 1.882–5.267); fluctuating type (had an OR = 6.234, 95%CI: 3.145–12.356); and worsening type had an (OR = 9.567, 95%CI: 4.823–18.952). All groups showed significantly increased odds of low cardiac rehabilitation participation, all *p* < 0.001.

After adjusting for sociodemographic characteristics in Model 2, and further adjusting for clinicopathological parameters and cardiac rehabilitation participation at T1 in Model 3, the final results showed that: persistently moderate-stress type had an (OR = 2.845, 95%CI: 1.542–5.248); persistently high-stress type had an (OR = 10.237, 95%CI: 5.128–20.415); remitting type had an (OR = 2.156, 95% CI: 1.298–3.582); fluctuating type had an (OR = 4.521, 95% CI: 2.567–7.965); and worsening type (had an OR = 6.834, 95% CI: 3.245–14.385). All groups still had significantly increased odds of low cardiac rehabilitation participation, all *p* < 0.05. The stress gradient followed the pattern of “persistently high stress > worsening > fluctuating > persistently moderate psychological stress > remitting > persistently low stress”, as detailed in Table 5.

**Table 5:** Association between acute psychological stress evolution trajectories and low cardiac rehabilitation participation in patients with acute myocardial infarction.

Models	Shift mode	B	SE	OR	95%CI	*p*-value
Model 1	Persistently low-stress (C1 → C1 → C1)	–	–	1.000 (Ref)	–	–
	persistently moderate-stress (C2 → C2 → C2)	1.417	0.325	4.124	2.156–7.892	<0.001
	Persistently high-stress (C3 → C3 → C3)	2.787	0.342	16.237	8.245–31.987	<0.001
	remitting (C3 → C2 → C1)	1.150	0.261	3.156	1.892–5.267	<0.001
	Fluctuating (C2 → C3 → C2)	1.830	0.351	6.234	3.145–12.356	<0.001
	Worsening (C1 → C2 → C3)	2.258	0.348	9.567	4.823–18.952	<0.001
Model 2	Persistently low-stress (C1 → C1 → C1)	–	–	1.000 (Ref)	–	–
	Persistently moderate-stress (C2 → C2 → C2)	1.218	0.338	3.381	1.742–6.563	0.002
	Persistently high-stress (C3 → C3 → C3)	2.521	0.365	12.445	6.078–25.478	<0.001
	remitting (C3 → C2 → C1)	0.982	0.272	2.669	1.568–4.545	0.003
	Fluctuating (C2 → C3 → C2)	1.625	0.332	5.078	2.648–9.735	<0.001
	Worsening (C1 → C2 → C3)	2.045	0.386	7.732	3.628–16.477	<0.001
Model 3	Persistently low-stress (C1 → C1 → C1)	–	–	1.000 (Ref)	–	–
	Persistently moderate-stress (C2 → C2 → C2)	1.045	0.315	2.845	1.542–5.248	0.012
	Persistently high-stress (C3 → C3 → C3)	2.326	0.351	10.237	5.128–20.415	<0.001
	remitting (C3 → C2 → C1)	0.768	0.258	2.156	1.298–3.582	0.006
	Fluctuating (C2 → C3 → C2)	1.508	0.288	4.521	2.567–7.965	<0.001
	Worsening (C1 → C2 → C3)	1.922	0.376	6.834	3.245–14.385	<0.001

Model 1: uncorrected; Model 2: adjusted for age, gender, marriage, education, occupation, and income; Model 3: The number of comorbidities, number of stents, LVEF, cardiac function classification and T1 cardiac rehabilitation participation were adjusted on the basis of model 2.

## Discussion

### Dynamic evolution of the prevalence of severe acute stress symptoms in patients with acute myocardial infarction after PCI

The results of this study showed that the prevalence of severe acute stress symptoms in patients with acute myocardial infarction after PCI exhibited a significant staged decreasing trend: 58.87% at T1, 29.84% at T2, and further decreased to 18.15% at T3. This trend is consistent with the conclusion of Ginty et al. (16), which indicates that after the acute phase of the disease, with the gradual recovery of physiological function, reconstruction of disease cognition and intervention of social support, acute stress symptoms in most patients can achieve observed reduction. However, it is worth noting that nearly one-fifth of patients still remained in a severe stress state at T3, suggesting that some patients face the stress of prolonged stress response (17).

In this study, data at T1 were collected via face-to-face paper questionnaires, while T2 and T3 data were collected through online questionnaires. The shift in data collection mode coincided exactly with the patients’ discharge period. Although we adopted unified instructions and telephone explanations to control for comprehension bias, we still cannot completely rule out the partial influence of measurement mode effects (e.g., differences in response environment and item comprehension) on the observed decline in stress levels. Future studies are advised to adopt a unified data collection method or to verify the measurement equivalence across different modes.

In addition, during the observation period of this study, all three centers provided patients with standardized discharge guidance and cardiac rehabilitation health education (including routine content such as medication guidance, exercise rehabilitation, dietary adjustments, and psychological adaptation), and no additional research-specific interventions were applied. This context suggests that the overall decline in stress levels from T1 to T3 may be partly attributable to the health education delivered as part of routine care, which helped patients develop a correct understanding of their disease (17–19). However, even under the coverage of routine care, 18.15% of patients still exhibited severe acute stress symptoms at T3, and significant differences in cardiac rehabilitation participation were observed among patients with different stress evolution trajectories. This fully indicates that routine care cannot completely replace precise, individualized interventions targeting high-risk psychological stress trajectories.

The innovation of this study is the addition of follow-up measurement at T3 (3 months after surgery), which reveals the complete evolution trajectory of stress from the acute phase to the mid-recovery phase. Univariate analysis further showed that stress-associated factors changed significantly across different stages: At T1, acute physiological stress indicators (cardiac function classification, number of stents, comorbidity burden) were dominant; socioeconomic and chronic health indicators (family monthly income, LVEF) began to show their influence at T2; at T3, family monthly income, number of comorbidities and cardiac function indicators still had independent predictive effects (20, 21). This finding suggests that clinical psychological intervention needs to adopt a staged and precise strategy.

### Profile characteristics of three categories of acute psychological stress in patients with acute myocardial infarction

In this study, latent profile analysis was used to identify three categories of heterogeneous structure of acute psychological stress in Chinese AMI patients, namely the low psychological stress group (C1), moderate psychological stress group (C2) and high psychological stress group (C3). Compared with the previous two-category classification, the three-category model can more accurately characterize the subgroup of patients with moderate psychological stress levels, and avoids information loss caused by simple dichotomization of a highly heterogeneous group. Changes in category probability across the three time points showed that the proportion of C3 decreased from 40 to 20%, the proportion of C1 increased from 21 to 52%, and the proportion of C2 remained between 28 and 39%, indicating that the moderate psychological stress group is a relatively stable but non-negligible middle population.

### Three-stage transition pattern of acute psychological stress in patients with acute myocardial infarction

This study identified six main evolution trajectories through three-stage latent transition analysis, which provides richer dynamic information than previous two-point studies. Among them, 28.23% of patients showed the persistently low-stress type (C1 → C1 → C1), which was the largest subgroup. Patients with the persistently high-stress type (C3 → C3 → C3) (22.18%) and the persistently moderate-stress type (C2 → C2 → C2) (18.55%) were the second and third largest subgroups, respectively. Together, these three subgroups accounted for 68.96% of the total sample, indicating that the stress state of most patients remained relatively stable during the observation period.

A total of 14.52% of patients showed a remitting trajectory (C3 → C2 → C1), that is, stress levels gradually decreased from high to low. This may be closely related to physical security, good family and social support, strong individual psychological resilience, and gradual improvement of cardiac function brought by restored blood flow after PCI (11). 8.47% of patients showed a worsening trajectory (C1 → C2 → C3), that is, stress levels gradually increased from low to high. This phenomenon of “delayed stress worsening” may occur because cognitive avoidance in the early stage of the disease masks real psychological distress. With realistic challenges after discharge, such as declining work ability, drug side effects, and forced lifestyle changes, accumulated psychological stress erupts in a concentrated manner (22, 23). 8.06% of patients had the fluctuating type (C2 → C3 → C2), with stress levels fluctuating back and forth between moderate and high, indicating that the stress response of some patients is unstable and requires continuous monitoring.

### Gradient association between acute psychological stress evolution trajectories and the odds of low cardiac rehabilitation participation

The core finding of this study is that there is a significant gradient association between different acute psychological stress evolution trajectories and the odds of low cardiac rehabilitation participation. Compared with the persistently low-stress type, the odds of low cardiac rehabilitation participation were significantly higher in the persistently moderate-stress type (OR = 2.845), persistently high-stress type (OR = 10.237), remitting type (OR = 2.156), fluctuating type (OR = 4.521), and worsening type (OR = 6.834). The gradient was characterized by the pattern of “persistently high-stress type > worsening type > fluctuating type > persistently moderate-stress type > remitting type”.

This gradient effect has clear clinical significance. First, due to the long-term high arousal state of persistently high-stress patients, negative emotions significantly weaken their willingness to participate in rehabilitation through the “motivation deprivation-cognitive bias” pathway (24, 25). Second, for patients with worsening trajectories, the process of shifting from low stress to high stress is often accompanied by the exhaustion of coping resources. Rehabilitation confidence established at the initial stage collapses amid practical setbacks, leading to rehabilitation avoidance behavior (26). The unstable stress state of patients with the fluctuating type makes it difficult to maintain continuous rehabilitation behavior. Even among patients who achieved stress relief, the stress of low rehabilitation participation was still about 2 times higher than that of the persistently low-stress type, suggesting that previous high stress exposure may have lasting effects on health behavior patterns.

The core finding of this study is that there is a significant gradient association between different acute psychological stress evolution trajectories and the odds of low cardiac rehabilitation participation. Compared with the persistently low-stress type, the odds of low cardiac rehabilitation participation were significantly higher in the other trajectory groups. The gradient was characterized by the pattern of “persistently high-stress type > worsening type > fluctuating type > persistently moderate-stress type > remitting type”. Importantly, due to the observational nature of this study, these findings reflect associations rather than causal effects.

## Limitations

This study has the following limitations:

Convenience sampling was used, and the sample was drawn from three tertiary A hospitals in Jiangsu Province, which may limit the generalizability of the findings.

The follow-up period was limited to 3 months after PCI, which precluded the observation of longer-term stress evolution trajectories.

All data were based on self-report scales, which may be subject to reporting bias. Additionally, the different modes of administration (face-to-face at T1 vs. online at T2 and T3) may have introduced measurement bias.

Although multivariate adjustments were performed, unmeasured confounding factors may still exist, and the observational design precludes the establishment of causal relationships.

Eight participants were lost to follow-up. Even though we supplemented the missing data through telephone follow-up, this may still have exerted a certain association.

Future studies should conduct multicenter, large-sample longitudinal studies with unified administration methods to further validate these findings.

## Conclusion

In this study, the three-stage latent transition model confirmed that there are six main dynamic evolution trajectories of acute psychological stress in AMI patients after PCI: 28.23% persistently low-stress type, 18.55% persistently moderate-stress type, 22.18% persistently high-stress type, 14.52% remitting type, 8.06% fluctuating type and 8.47% worsening type. There was a significant gradient association between different stress trajectories and cardiac rehabilitation participation, and the odds of low rehabilitation participation in each trajectory group were significantly higher than those in the persistently low-stress type. Clinically, a closed-loop, full-cycle psychological management framework based on the three-class profiles should be established, incorporating early identification, dynamic monitoring, and stratified intervention, with a particular focus on patients along the three trajectory types: persistently high-stress, worsening, and fluctuating types. These groups may warrant closer psychological monitoring and further evaluation in future intervention studies, to verify whether targeted interruption of the persistently and worsening stress pathways is associated with enhanced cardiac rehabilitation participation.

## Data Availability

The original contributions presented in the study are included in the article/supplementary material, further inquiries can be directed to the corresponding author/s.
